# Supplementary material for: The Tunable Electronic and Optical Properties of Two-Dimensional Bismuth Oxyhalides
Source: Nanomaterials (Basel). 2023 Oct 20;13(20):2798. doi: 10.3390/nano13202798 (PMC10609128; doi:10.3390/nano13202798)
Supplement: Supplementary file 1 [file nanomaterials-13-02798-s001.zip › nanomaterials-2630772-supplementary.pdf]

Table S1. The calculated lattice parameters constants *a* (Å) of BiOX (X = Cl, Br, I, $Cl_{0.5}Br_{0.5}$, $Br_{0.5}I_{0.5}$, $Cl_{0.5}I_{0.5}$) monolayer.

System	BiOCl	$BiOCl_{0.5}Br_{0.5}$	BiOBr	$BiOCl_{0.5}I_{0.5}$	$BiOBr_{0.5}I_{0.5}$	BiOI
a (Å)	3.876	3.900	3.926	3.945	3.970	4.015
Ref.[39] (Cal.)	3.889		3.930			4.014
Ref.[23] (Cal.)	-		-			4.020
Ref.[40] (Exp.)	3.890		3.916			3.985

Table S2. The calculated HSE06-based band gaps of BiOX (X = Cl, Br, I) monolayer.

System	BiOCl	BiOBr	BiOI
Band gap (eV)	3.745	3.354	2.278
Ref.[39] (Cal.)	3.79	3.41	2.3
Ref.[23] (Cal.)	-	-	2.28

Table S3. The variation of calculated static dielectric constant $\varepsilon(0)$ and static refractive index $n(0)$ of BiOX (X = Cl, Br, I) with the layer numbers.

System	BiOCl		BiOBr		BiOI	
	$\varepsilon(0)$	$n(0)$	$\varepsilon(0)$	$n(0)$	$\varepsilon(0)$	$n(0)$
1 Layer	1.70	1.30	1.83	1.35	2.15	1.47
2 Layers	2.23	1.49	2.41	1.55	3.08	1.75
3 Layers	2.49	1.58	2.78	1.67	3.42	1.85
4 Layers	2.81	1.68	3.17	1.78	3.98	1.99
5 Layers	2.93	1.71	3.28	1.81	4.26	2.06
Bulk	4.41	2.10	4.96	2.23	6.22	2.49

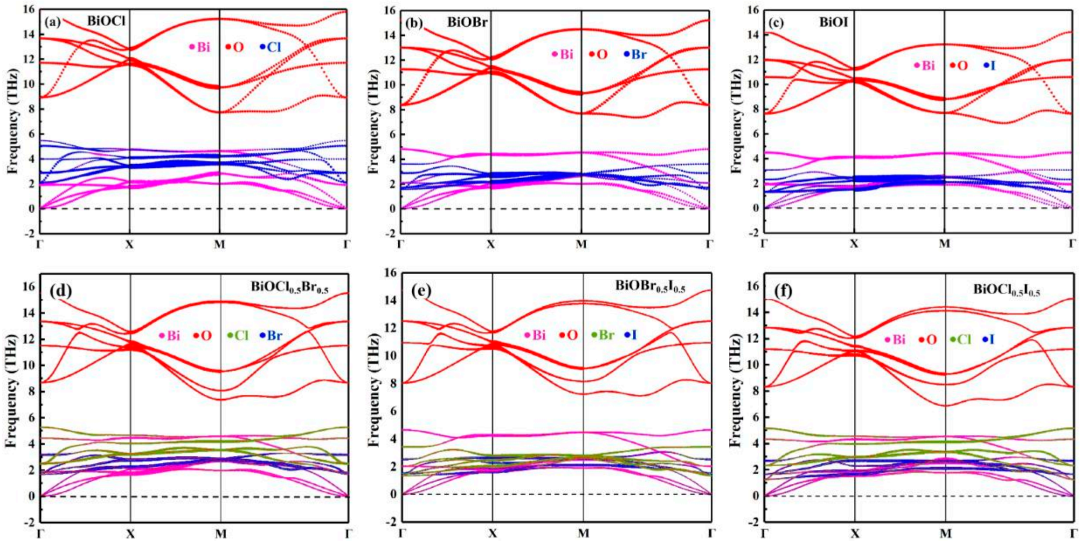

Figure S1. (a–f) The phonon spectra of the BiOX (X = Cl, Br, I, $Cl_{0.5}Br_{0.5}$, $Br_{0.5}I_{0.5}$, $Cl_{0.5}I_{0.5}$) monolayer. Γ–X–M–Γ are the high symmetric points.

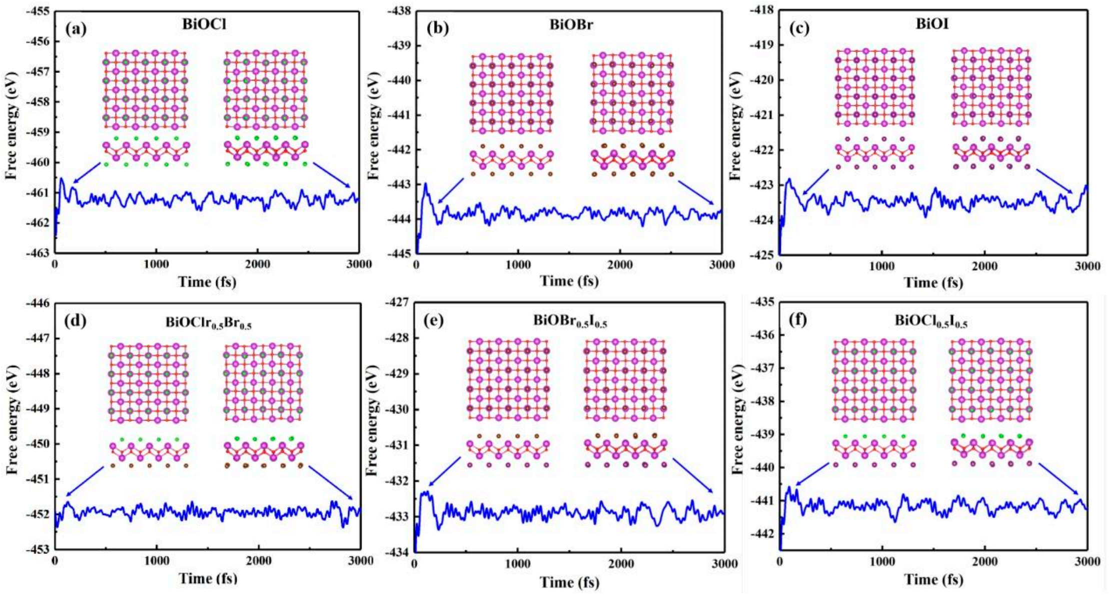

Figure S2. (a−f) The free energy variation in the AIMD simulation at constant temperature (300K) of the BiOX (X = Cl, Br, I, $Cl_{0.5}Br_{0.5}$, $Br_{0.5}I_{0.5}$, $Cl_{0.5}I_{0.5}$) monolayer.

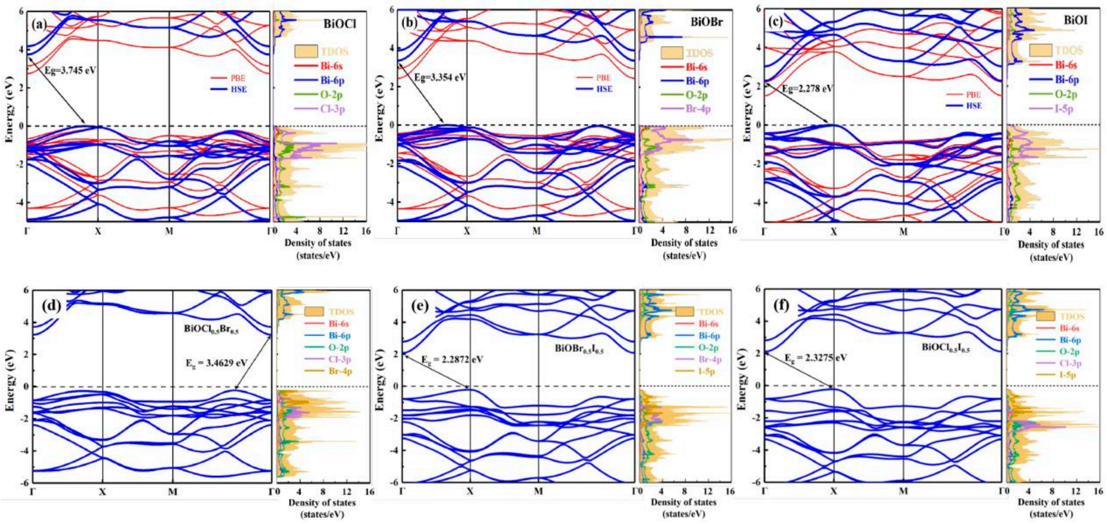

Figure S3. Left panels of (a−f): the band structures of the BiOX (X = Cl, Br, I, $Cl_{0.5}Br_{0.5}$, $Br_{0.5}I_{0.5}$, $Cl_{0.5}I_{0.5}$) monolayers along the high-symmetry lines Γ−X−M−Γ; Right panels of (a−f): the total and projected DOS of the BiOX (X = Cl, Br, I, $Cl_{0.5}Br_{0.5}$, $Br_{0.5}I_{0.5}$, $Cl_{0.5}I_{0.5}$) monolayer.

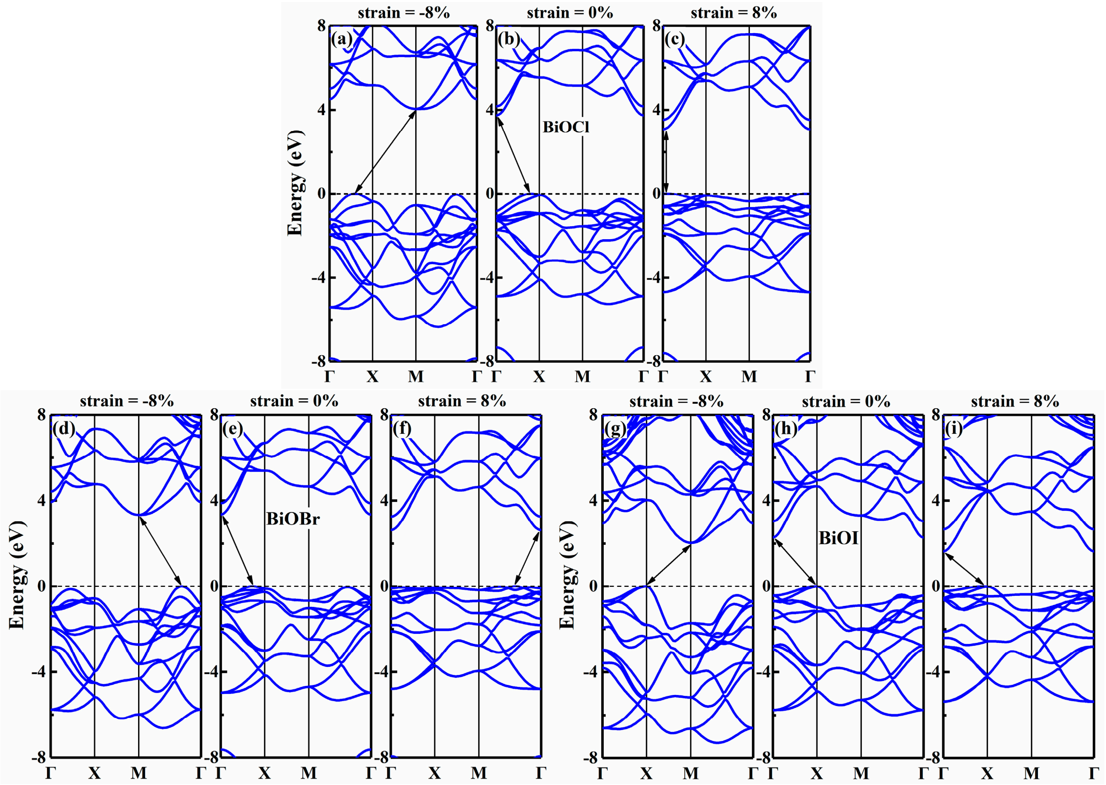

Figure S4. Band structures of (a)(b)(c) BiOCl, (d)(e)(f) BiOBr, and (g)(h)(i) BiOI monolayers under biaxial strain at −8%, 0% and 8%.
